# UIO-66 as Nucleating Agent on the Crystallization Behavior and Properties of Poly(Ethylene Terephthalate)

**DOI:** 10.3390/polym13142266

**Published:** 2021-07-10

**Authors:** Yue Yin, Yuan Wang, Linghui Meng

**Affiliations:** 1 Department of Polymer Materials and Engineering, School of Chemistry and Chemical Engineering, Harbin Institute of Technology, Harbin 150001, China; yinyue@stu.hit.edu.cn; 2 Department of Building Science, School of Architecture, Beijing Key Laboratory of Indoor Air Quality Evaluation and Control, Tsinghua University, Beijing 100084, China; dr@yuanwang.name

**Keywords:** polyethylene terephthalate, crystallization, nucleating agent, MOFs

## Abstract

In this study, not only was the similar terephthalate structure between UIO-66 and PET utilized to improve compatibility, but the Zr^4+^ exposed by defects of UIO-66 was also utilized to improve the interaction between PET and UIO-66. Furthermore, PET nanocomposites with different contents of UIO-66 were also fabricated. Due to the high specific surface area and coordination of Zr^4+^, UIO-66 has high nucleation efficiency in the PET matrix. Compared with pure PET, the crystallization rate of PET/UIO-66 nanocomposite is significantly increased, and the crystallization temperature of PET-UIO66-1 is significantly increased from 194.3 °C to 211.6 °C. In addition, the tensile strength of nanocomposites has also been improved due to coordination.

## 1. Introduction

Poly(ethylene terephthalate) (PET), a thermoplastic semi-crystalline polymer [1], has been extensively employed in the industrial applications of packaging materials, optical materials and engineering plastics, due to its prominent physical and chemical properties (e.g., high strength and high thermal stability) [2,3]. Though PET molecules exhibit a symmetrical linear structure, the crystallization rate remains low because of the rigid benzene ring structure in the molecular chain. The slow crystallization rate seriously limits the application of the PET in engineering plastics fields [4], especially the manufacture of injection molded products. On the whole, such products with PET as the raw material require longer molding cycles and higher mold temperatures to be well prepared [5,6]. Accordingly, the improvement of the PET crystallization rate has always been a research hotspot in the application of PET to structural materials [7,8,9].

Heterogeneous nucleation refers to a method broadly applied by researchers to elevate the crystallization rate of polymers. The nucleating agent aims to implement the principle of heterogeneous nucleation by introducing foreign matters into a polymer matrix to replace the nucleation of the polymer, thereby effectively facilitating the crystallization and reducing the crystal size. Conventional inorganic nucleating agents (e.g., silica [10,11,12], titanium dioxide [13], mica [14], clay [15,16] and glass flask [17]) exhibit a poor compatibility with the PET matrix. Moreover, organic small molecule sodium salts’ nucleating agents (e.g., sodium benzoate [18]) may cause the degradation of the PET matrix during processing [19]. Thus, the above-mentioned two kinds of nucleating agents exhibit their own defects. Over the past few years, the synthesis or modification of inorganic materials to prepare organic–inorganic hybrid nucleating agents turns out to have aroused the most attention from researchers. Polyhedral oligomeric silsesquioxane (POSS) [20], modified graphene oxide [21,22] and organic silica quantum dots [23] have been developed to up-regulate the crystallization rate of PET due to their excellent compatibility with the PET matrix. As reported in the existing studies, the increase in the specific surface area helps improve the nucleation efficiency of the nucleating agent [24]. However, the common modification method of inorganic particles is to form a core–shell structure by covering a layer of organic materials on the surface of inorganic ions. Although such a method can improve the compatibility between PET and inorganic particles, the specific surface area of the nucleating agent is narrowed, thereby hindering the further improvement of nucleation efficiency.

Metal–organic frameworks (MOFs) refer to a type of organic–inorganic hybrid material coordinated with organic linkers, with inorganic metal as the nodes. Over the past decade, MOFs have been extensively studied as a porous material for their ultra-high specific surface area and porous structure. Among a wide range of MOFs, the Zr-containing MOF, UIO-66(Zr), developed by the University of Oslo [25], has aroused widespread attention for its excellent solvent resistance and high thermal stability. UIO-66(Zr) is formed by 12-coordination between the Zr_6_O_4_(OH)_4_ unit and terephthalic acid ligand [26], so it is considered to exhibit a high compatibility with PET. Existing studies have been broadly conducted to prepare UIO-66 from terephthalic acid, which is obtained by degrading waste PET [27,28]. According to the knowledge of the authors, there have been some constructional reports of MOFs used as the nucleating agents in the PET composites. Among them, Dominici et al. [29] innovatively prepared terephthalate calcium salts and added them to PET to prepare nanocomposites to form a Ca–metal–organic framework to improve the mechanical properties of the composites. This finding indicated that MOFs and coordination have a significant impact on the performance of the PET matrix. 

In this study, to fully exploit the ultra-high specific surface area and the terephthalate structure of UIO-66, UIO-66 was introduced as a nucleating agent into the PET matrix, and PET nanocomposites with different contents of UIO-66 were prepared. Besides, the effects of UIO-66 exhibiting organic–inorganic hybrid structures on the crystallization rate and crystallization behavior of PET, the PET matrix structure, and its thermal and mechanical properties, were analyzed. Furthermore, under non-isothermal conditions, the nucleation and crystallization behavior exhibited by the PET nanocomposites were studied in depth.

## 2. Materials and Methods

### 2.1. Materials

**Figure 1:**
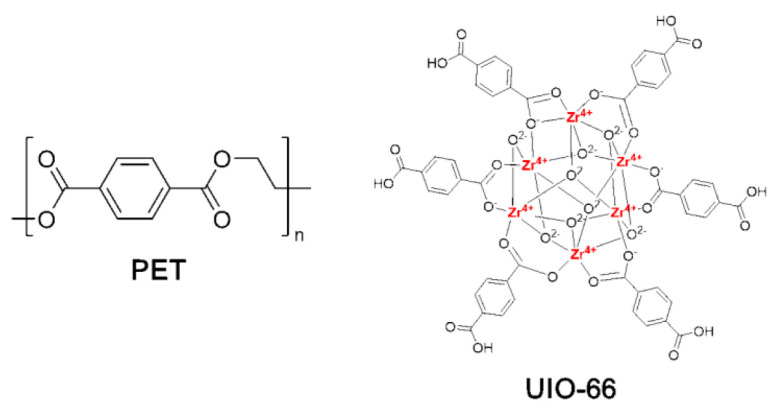
Structure of PET and UIO-66(Zr).

Poly(ethylene terephthalate) ([η] = 0.68 g/dL) and UIO-66(Zr) (chemical structures illustrated in Figure 1) were prepared in the laboratory of the authors’ research group. PET was synthesized according to the method of our previous work [6]. UIO-66(Zr) was synthesized according to a method given in the the literature [30]. 1,1,1,3,3,3-Hexafluoroisopropanol (HFiP) originated from Langhua Chemical Co., Ltd. (Shandong, China).

All the chemicals were directly employed without being further purified.

### 2.2. Experiments

Prior to the experiments, PET was placed in a constant-temperature blast drying oven at 80 °C and then dried for 4 h. Five grams of PET with different mass fractions of UIO-66 (based on the weight of PET, and the UIO-66 added at 0.5 wt%, 1 wt%, and 3 wt%) was dissolved in 40 mL of HFiP. In addition, the mixture was stirred at 50 °C for 4h. Afterwards, the solution was poured into a Teflon tray to volatilize the solvent overnight at ambient temperatures. After being dried in an oven at 80 °C for 24 h, the obtained solid was transferred to a vacuum drying oven at 80 °C for 24 h to further remove the solvent. The resulting solid was crushed into powder with a high-speed crusher. Next, the powder was marked as PET-UIO66-0.5, PET-UIO66-1, and PET-UIO66-3, respectively.

The preparation of the PET nanocomposite films is elucidated below. The powders of PET with UIO-66 were dried in a constant-temperature oven at 80 °C for 4 h. Subsequently, the dried powder was placed between two polyimide films. The films were hot-pressed within a constant-temperature heating plate for 3 min at 280–290 °C. Next, the air bubbles were extracted by adopting a scraper in the hot-pressing. Afterwards, the films were quenched in ice water, and then the polyimide films were peeled off to generate the PET/UIO-66 nanocomposites. The films exhibited the thickness of nearly 100 μm.

### 2.3. Characteristics

UIO-66 and its dispersion in PET nanocomposites were observed using a scanning electron microscope (JSM-IT300, JEOL, Tokyo, Japan), and the accelerating voltage was 15 kV. The samples were cryofractured in liquid nitrogen, and then vacuum-coated with a layer of platinum.

The thermal stability of the PET/UIO-66 nanocomposites were tested with Q500 thermogravimetric analyzer (TA, New Castle, DE, USA). Approximately 8mg of the sample was placed in a platinum basket. Subsequently, the temperature was raised from the ambient temperature to 550 °C at a heating rate of 10 °C/min in an argon atmosphere, and the weight loss curves were plotted.

The crystallization behavior of the PET/UIO-66 nanocomposites was examined with Q20 Differential Scanning Calorimeter (TA, New Castle, DE, USA). About 8 mg of the sample was added to the aluminum crucible with a lid and then placed into the equipment to be tested. The respective sample was heated to 280 °C at a heating rate of 10 °C/min, and then it stayed for 5 min to eliminate the thermal history. Next, the sample was cooled to 30 °C at a cooling rate of 10 °C/min. Lastly, the sample was heated to 280 °C at a heating rate of 10 °C/min. Moreover, the non-isothermal crystallization properties of the PET/UIO-66 nanocomposites were characterized by using Q20 differential scanning calorimeter (TA, USA). Nearly 8 mg of the sample was added to the aluminum crucible with a lid and then placed into the equipment to be tested. The respective sample was heated to 280 °C at a heating rate of 40 °C/min, and then it stayed for 5 min to eliminate the thermal history. Subsequently, the samples were cooled to 30 °C at a cooling rate of 5, 10, 15 and 20 °C/min, and the non-isothermal crystallization was recorded. The whole experiment was performed under a nitrogen atmosphere, and the gas flow rate reached 50 mL/min.

The degree of crystallinity of the samples was calculated as:

(1) $$X_{c}=\frac{\Delta H_{m}}{w\times \Delta H_{m}^{0}}\times 100\%$$

where $\Delta H_{m}$ denotes the melting enthalpy of the PET; $\Delta H_{m}^{0}$ represents the melting enthalpy of the 100% crystalline PET ($\Delta H_{m}$ 100%); *w* expresses the weight fraction of the PET in the nanocomposite. The melting enthalpy (Δ*Hm* 100%) of the 100% crystalline PET was reported in the literature as 140 J/g [31,32].

A three-phase model is used to analyze the high-order structure of PET, including crystalline phase, rigid amorphous fraction (RAF) and movable amorphous fraction (MAF). The MAF content of the PET nanocomposite was calculated by the heat capacity change at the glass transition temperature. The *X*_MAF_ and *X*_RAF_ in nanocomposites was calculated by [29,33]:

(2) $$X_{\mathrm{MAF}}=\frac{\Delta C_{p}}{\Delta C_{p}^{0}}\times 100\%,$$

(3) $$X_{\mathrm{RAF}}=1-X_{c-}X_{\mathrm{MAF}}$$

where $\Delta C_{p}$ denotes the heat capacity change at *T*_g_ of PET nanocomposites; $\Delta C_{p}^{0}$ represents the heat capacity change at *T*_g_ of the 100% amorphous PET. The $\Delta C_{p}^{0}$ of the 100% amorphous PET was reported in the literature as 0.405J/(g × °C) [34].

The tensile strength data of the PET/UIO-66 nanocomposites were acquired with a universal stretching machine (Instron, Norwood, MA, USA). Next, the PET/UIO-66 nanocomposites films were cut into the tensile specimens by using dogbone-shaped cutters. The sample size was 35 mm × 2 mm dogbone-shaped sample, and the stretching speed was 10mm/min. Each sample was run for five times and all the reported tensile properties were averages of these tests.

The WAXD patterns of the UIO-66 and the PET/UIO-66 nanocomposites were collected on an Empyrean Panalytical X-ray diffractometer (Malvern panalytical, Almelo, The Netherlands). The sample films were fixed on a smooth silicon plate to ensure the sample to be flat for testing. The 2θ scan range was 5° to 60°.

## 3. Results and Discussions

### 3.1. Dispersion of UIO66 within PET/UIO-66 Nanocomposites

**Figure 2:**
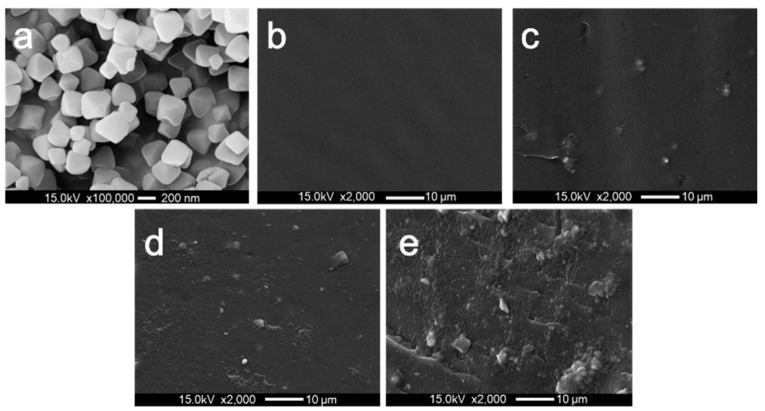
SEM image of PET and PET/UIO-66 nanocomposites: (**a**) UIO-66; (**b**) neat PET, (**c**) PET-UIO66-0.5; (**d**) PET-UIO66-1 and (**e**) PET-UIO66-3.

The dispersion of the nucleating agent refers to one of the critical factors for the efficiency of the polymer crystallization nucleation. To investigate the dispersion of UIO-66 in the PET matrix, the dispersion morphologies of the PET and the PET/UIO-66 nanocomposites were analyzed by using the SEM images. As indicated from the SEM images in Figure 2b–e, for the samples exhibiting different UIO-66 contents from 0 to 3 wt%, UIO-66 was observed to be dispersed in the PET matrix in the micrometer range. It was, therefore, suggested that when the addition amount was only 0.5–1%, UIO-66 exhibited a high dispersibility in the PET matrix. However, as the addition amount further increased to 3%, UIO-66 was obviously agglomerated in the PET matrix.

### 3.2. Melting Behavior and Crystallization of the PET/UIO-66 Nanocomposites

**Figure 3:**
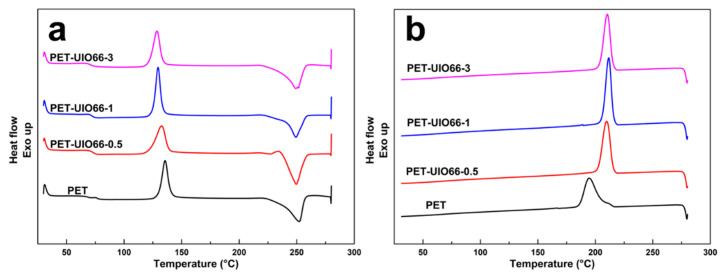
DSC thermograms of the PET/UIO-66 nanocomposites: (**a**) first heating scans and (**b**) cooling scans.

The non-isothermal DSC test was performed to characterize the effect of the UIO-66 content on the crystallization properties of the PET/UIO-66 nanocomposites. The relevant DSC curve is plotted in Figure 3. The data measured by DSC, including the glass transition temperature (*T*_g_), cold crystallization temperature (*T*_cc_), melting temperature (*T*_m_), crystallization temperature (*T*_c_) and related enthalpies (Δ*H*_cc_, Δ*H*_m_ and Δ*H*_c_), are listed in Table 1. In Figure 3a, both the pure PET and the PET/UIO-66 nanocomposites showed cold crystallization peaks on the first heating scan. As the content of UIO-66 increases, the cold crystallization temperature tends to decrease. As indicated from the figure, UIO-66 could facilitate the formation of crystals in the amorphous region during heating. As suggested by the first cooling curve (Figure 3b), as the addition of UIO-66 increased to 1%, *T*_c_ increased significantly from 194.3 °C to 211.6 °C, thereby indicating that UIO-66 could significantly promote the crystallization of PET. However, with the increase in the content of UIO-66, the crystallization temperature decreased, which demonstrated that as the content of UIO-66 increased, the folding and rearrangement of the PET molecules would be inhibited. According to the results, UIO-66 could act as a nucleating agent to form heterogeneous nucleation points in the cooling of the melt, and a lower content of UIO-66 was sufficient as a nucleating agent for PET to achieve heterogeneous nucleation.

**Figure 4:**
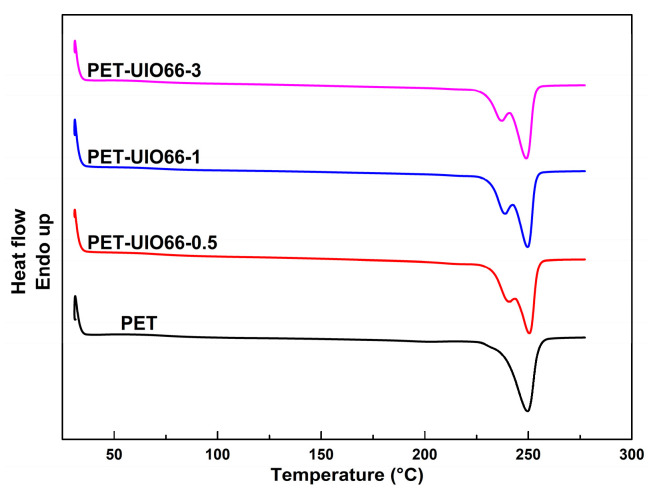
Second heating scans of the PET and the PET/UIO-66 nanocomposites.

According to Figure 4, the second heating scan was performed at 10 °C/min. Table 2 lists the relevant characteristic data. In the second heating, the PET and the PET/UIO-66 nanocomposites showed insignificant glass transition temperature. This was because the PET exhibited a high crystallinity at the previous cooling rate of 10 °C/min. Thus, the glass transition did not vary significantly in terms of the heat capacity of the material, and a step transition could be difficult to observe. The melting crystallization was relatively sufficient, and the cold crystallization peak was not easy to observe. Moreover, for all the PET/UIO-66 nanocomposite samples, the DSC endothermic peaks displayed the double peaks. For the pure PET, only one melting peak was identified at 249.7 °C. Although the PET samples had identical thermal histories, only the double melting peaks were identified in the PET/UIO-66 nanocomposites samples, which might be attributed to a small amount of imperfect crystals in the composites caused by the nucleating agent in the rapid cooling. Unstable crystals (imperfect lamellae) melt through heating, and then the molecular chain moves and recrystallizes into a more perfect crystal, which melts at a higher temperature. Accordingly, a double melting peak was identified in the second heating curve of the PET/UIO-66 nanocomposite.

**Figure 5:**
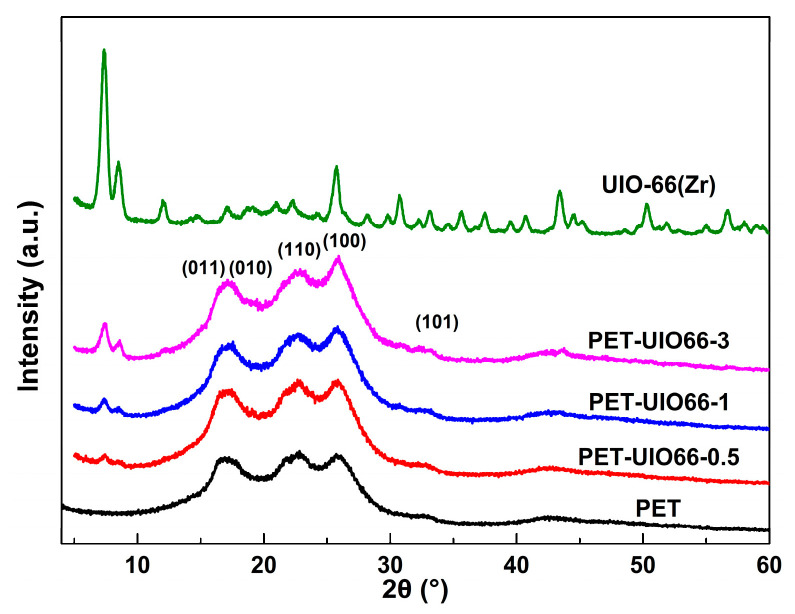
XRD pattern of the UIO-66, the PET and the PET/UIO-66 nanocomposites.

Given the wide-angle X-ray diffraction, the effect of UIO-66 on the crystal structure of the PET matrix was studied. According to Figure 5, for the PET crystal diffraction pattern, the characteristic diffraction peaks were at 17.5° (010), 22.7° (110), 25.8° (100) and 32.8° (101), respectively. Likewise, all patterns of the PET/UIO-66 nanocomposites appeared at four diffraction peaks in identical positions, which demonstrated that the introduction of UIO-66 into the PET matrix would not convert the original crystal structure of PET. The XRD pattern of the PET-UIO66-3 showed sharper diffraction peaks. It was, therefore, suggested that the nucleation effect of UIO-66 could significantly increase the crystallization rate of PET and gradually perfect the PET crystallite [35]. It is noteworthy that the three samples of the PET/UIO-66 nanocomposites displayed the characteristic diffraction peaks of UIO-66 at 7.4° and 8.5° [36]. The intensity of UIO-66′s characteristic diffraction peaks increased significantly with the increase in the UIO-66 content.

### 3.3. Non-Isothermal Crystallization Kinetics of the PET/UIO-66 Nanocomposites

In the actual injection molding of engineering materials, the materials are generally in a non-isothermal cooling process. Thus, the crystallization kinetics of the PET/UIO-66 nanocomposites should be further assessed.

**Figure 6:**
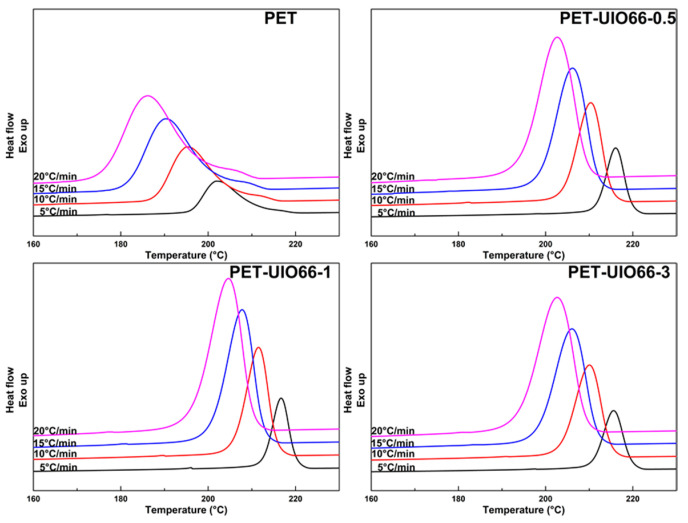
Non-isothermal crystallization curves of PET and PET/UIO-66 nanocomposites at different cooling rates.

According to Figure 6, the non-isothermal crystallization process of the PET and the PET/UIO-66 nanocomposites at 5, 10, 15 and 20 °C/min were studied. As the cooling rate increases, the crystallization temperature gradually decreases. The result is that the excessively fast cooling rate inhibits the formation of nuclei and the growth of crystals. In addition, it can be observed that the crystallization peak of the PET/UIO-66 nanocomposites is sharper and the crystallization temperature is higher at the same cooling rate. This improvement is attributed to the excellent heterogeneous nucleation property of UIO-66 in the PET matrix.

**Figure 7:**
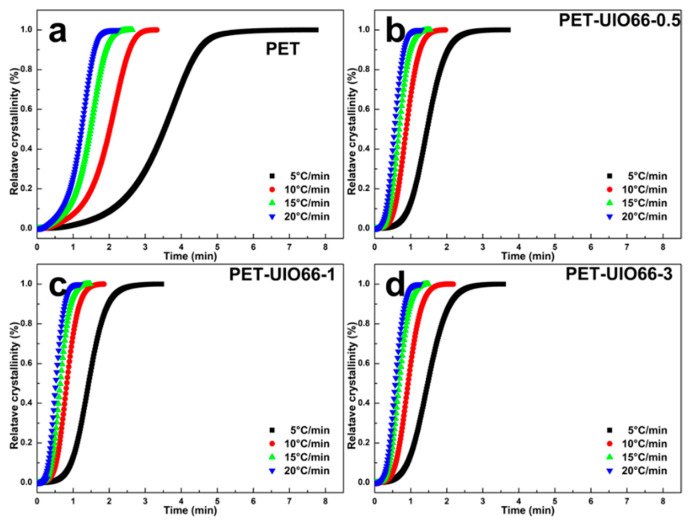
Relationship between time and relative crystallinity of the PET and the PET/UIO-66 nanocomposites at different cooling rates: (**a**) neat PET, (**b**) PET-UIO66-0.5; (**c**) PET-UIO66-1 and (**d**) PET-UIO66-3.

Figure 7 shows the relationship between the time vs. relative crystallinity curves of the PET and the PET/UIO-66 nanocomposites. According to the relative crystallinity curve, the crystallization time of the PET/UIO-66 nanocomposites is much shorter than that of the pure PET. Due to thermal hysteresis, the crystallization time of the PET and the PET/UIO-66 nanocomposites gradually decrease as the cooling rate increases. Compared with the pure PET, different cooling rates have less effect on the complete crystallization time of the PET/UIO-66 nanocomposites, thereby indicating that UIO-66 can exhibit strong heterogeneous nucleation properties in the PET matrix.

**Figure 8:**
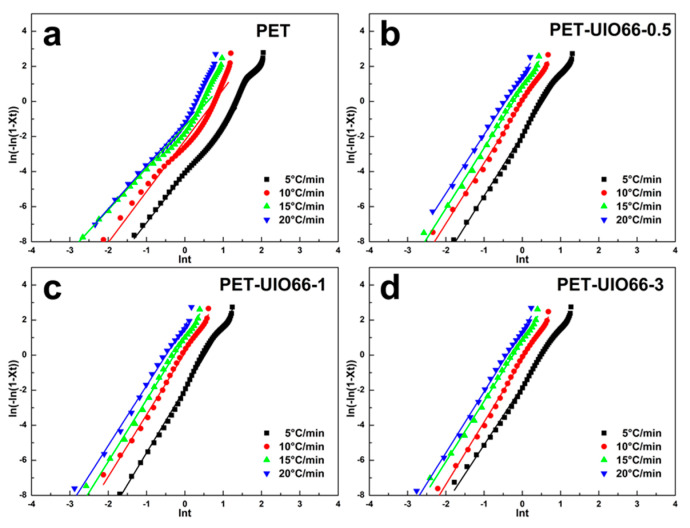
Non-isothermal crystallization kinetic of PET and PET/UIO-66 nanocomposites at different cooling rates: (**a**) neat PET, (**b**) PET-UIO66-0.5; (**c**) PET-UIO66-1 and (**d**) PET-UIO66-3.

Jeziomy’s method was adopted to calculate the non-isothermal crystallization kinetics of the PET/UIO-66 nanocomposites. Avrami index (n) and crystallization rate constant (Z_c_) were derived from Figure 8; the results are listed in Table 3. Zc denotes the crystallization rate parameter; the higher the Z_c_ value, the faster the crystallization would be. n expresses Avrami index, determining the growth behavior of the PET crystals. According to the non-isothermal crystallization data, under the identical cooling rate, with the increase in the UIO-66 content, Zc first increased and then decreased, reaching a peak value when the UIO-66 content was 1 wt%, which demonstrated that a small amount of UIO-66 would act as a nucleation point and promote the crystallization of PET. Although the physical meaning of n could not be simply connected with actual non-isothermal crystallization, it could express the growth trend of crystals in non-isothermal crystallization. The Avrami index of the pure PET is between 2 and 3, which demonstrated three-dimensional spherical growth. The Avrami index n of the PET/UIO-66 nanocomposites was between 3 and 4, which demonstrated spherical growth. As the UIO-66 content increased from 0.5 to 3%, n first increased and then decreased. It was further confirmed that though UIO-66 exerted a nucleation effect in the PET matrix, excessive UIO-66 limited the migration of molecular chains, to a certain extent, and inhibited the growth of three-dimensional crystals. As revealed from the above results, the introduction of a small amount of UIO-66 could increase the crystallization rate of PET. This discovery could be attributed to the high specific surface area of UIO-66, enhancing its own nucleation effect, as well as to the coordination of Zr4+ in UIO-66 with the carbonyl group in PET, improving the heterogeneous nucleation ability.

### 3.4. Thermal Stability of the PET and the PET/UIO-66 Nanocomposites

**Figure 9:**
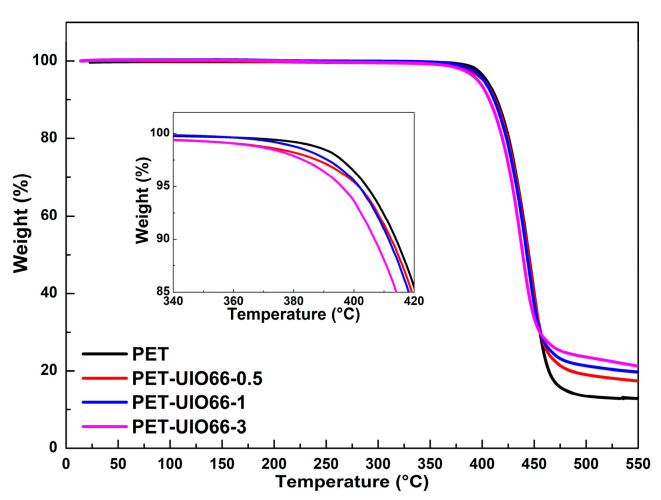
The TGA curves of the PET and the PET/UIO-66 nanocomposites.

Given the existing reports, the addition of some nucleating agents will seriously deteriorate the thermal stability of the matrix [37]. Accordingly, the effect of UIO-66 on the thermal stability of PET could act as an important indicator to assess the performance of the PET/UIO-66 nanocomposites. According to Figure 9, the thermal stability of the PET and the PET/UIO-66 nanocomposites was assessed by conducting the TG analysis in an inert atmosphere. Table 4 lists the specific initial decomposition temperature, maximum decomposition temperature and residues.

According to Table 4, with the increase in the UIO-66 content, the initial decomposition temperature of the PET/UIO-66 nanocomposites (*T*_5%_ and *T*_10%_, the temperature during weight loss reached 5% and 10%) slightly decreased. Many studies highlighted that defects usually exist in UIO-66 in the form of missing linkers [38,39]. Such a defect caused Zr^4+^ ions to be directly exposed in the PET matrix. When UIO-66 was introduced into the PET matrix, the exposed Zr^4+^ might catalyze the degradation of PET to a certain extent, thereby resulting in a small decrease in the initial decomposition temperature and the maximum decomposition temperature. Moreover, as revealed from the results, the residue of the nanocomposites increased significantly at 550 °C, and the residue increased from 13.2% for pure PET to 21.2% for PET-UIO66-3. Since UIO-66 exhibited a higher specific surface area, it could help to charge PET and increase the residue of the PET/UIO-66 nanocomposite. Thus, the residues of the PET-/UIO-66 nanocomposites increased significantly at 550 °C.

### 3.5. Mechanical Property of the PET and the PET/UIO-66 Nanocomposites

**Figure 10:**
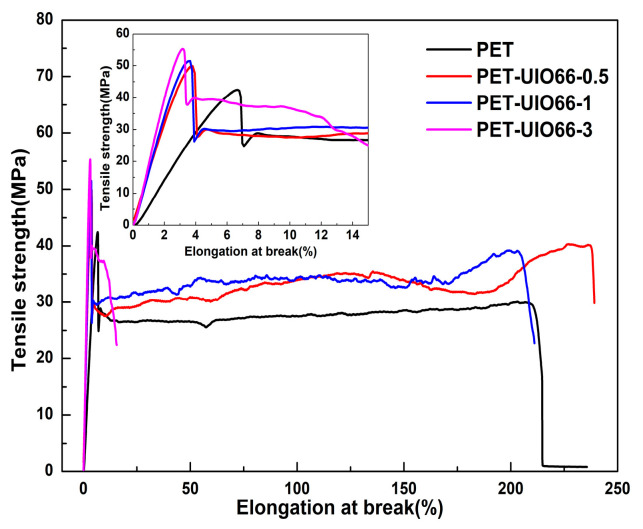
The stress–strain curves of PET and PET/UIO-66 nanocomposites.

Figure 10 plots the stress–strain curves of the PET and the PET/UIO-66 nanocomposites. Table 5 lists the specific characteristics of the tensile test. The elongation at break of PET-UIO66-0.5 and PET-UIO66-1 was consistent with that of pure PET. However, when the amount of UIO-66 added increased to 3%, the elongation at break of the PET dropped significantly. This was because considerable UIO-66 led to an agglomeration in the mixing process, thereby reducing the elongation at break of the material. This was also proved by SEM images presented in Figure 3e. Another reason was that the defects in UIO-66 exposed more Zr^4+^ to coordinate with the carbonyl group in the PET, thereby causing the increased rigidity and brittleness of the PET-UIO-66-3.

It was precisely due to the coordination effect of a small amount of Zr^4+^ with the PET matrix that the PET/UIO-66 nanocomposites exhibited a higher tensile strength and a greater tensile modulus. As indicated from the figure, with the increase in the amount of UIO-66 added, the tensile strength and tensile modulus of the PET composites increased significantly, which was attributed to the rigid structure of the UIO-66 and the coordination between Zr^4+^ and the PET molecules to expedite the interaction between the PET molecules.

### 3.6. Three-Phase Model of the PET and the PET/UIO-66 Nanocomposites

**Figure 11:**
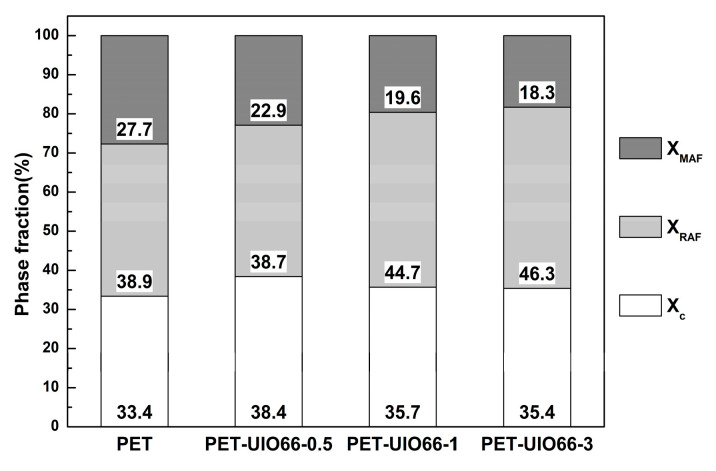
Different phase fractions of PET and the PET/UIO-66 nanocomposites.

UIO-66 will also produce the blocking effect of the amorphous phase due to the presence of coordination, resulting in an increase in the proportion of rigid amorphous regions. Therefore, UIO-66 has two different functions in PET/UIO66 nanocomposites: the nucleation effect of the nucleating agent and the blocking effect caused by coordination [29]. The three-phase model is illustrated in Figure 11 to explain the role of UIO-66 in the PET matrix. When the addition amount is low, the nucleation effect dominates and the crystallinity increases, while X_RAF_ is similar to pure PET. As the filler content further increases, the crystallization is mainly inhibited by the blocking effect from the coordination. The blocking effect plays a dominant role, causing the crystallinity to decrease instead, but X_RAF_ further increases. In the PET/UIO-66 nanocomposites, as the content of UIO-66 increases, the total content of X_c_ + X_RAF_ increases. This also helps explain the significant increase in the modulus of the nanocomposite in Figure 10.

### 3.7. Crystallization Mechanism of PET and PET/UIO-66 Nanocomposites

**Figure 12:**
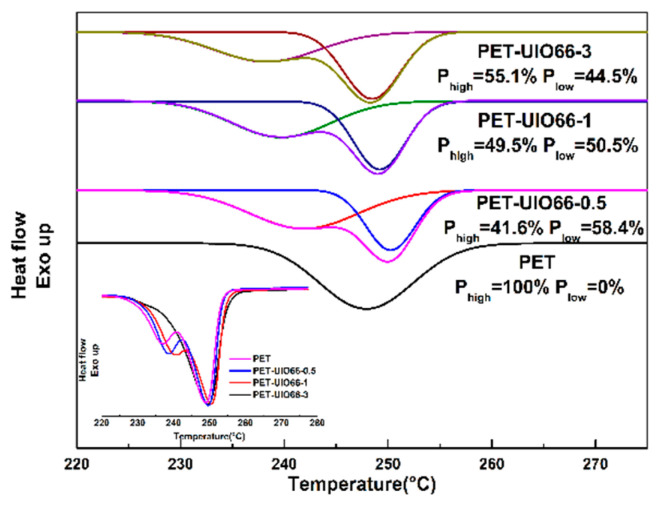
The original melting curve and the second heating process fitting curve of the PET and the PET/UIO-66 nanocomposites.

The second heating process of the PET nanocomposite was fitted, and the proportion of the peaks in the melting process of the different melting points were also investigated. The specific results are shown in Figure 12. It can be seen that after UIO-66 was added, a melting peak with a lower melting point (P_low_) appears. With the further increase in UIO-66 content, the proportion of P_low_ in the total melting peak gradually decreases. However, it can be seen from the enlarged melting curves that although there are obvious double peaks during the melting process, the area of the peak of the higher melting point (P_high_) does not change significantly. This shows that UIO-66 as a nucleating agent can not only promote the crystallization rate of PET, but also form more imperfect crystals in the PET matrix. These imperfect crystals lead to the formation of double melt peaks in the nanocomposites. However, with the further increase in the content of UIO-66, the restriction, caused by Zr^4+^, of the molecular movement ability of PET nanocomposites becomes stronger and, therefore, the number imperfect crystals is reduced. This also confirms the conclusion of the three-phase model in the previous section: as the content of UIO-66 increases, the coordination of the carboxyl group in the PET molecules and Zr^4+^ in UIO-66 restricts the movement of the PET molecular chain so that X_RAF_ increases and X_c_ decreases.

## 4. Conclusions

In this study, the PET/UIO-66 nanocomposites containing UIO-66 were prepared by solvent blending. The thermal performance of the nanocomposites was slightly lower than that of the PET, whereas the residue at 550 °C was significantly improved. In the nanocomposite film containing 1% UIO-66 by weight, the tensile strength increased by 21.3%, and the elongation at break was consistent with that of the pure PET. In the composites with a content of 3% the UIO-66, the tensile strength increased to as much as 57.6 MPa, an increase of 31.0%. Notably, the addition of the UIO-66 significantly increased the crystallization temperature of PET from 194.3 °C for pure PET to 211.6 °C for PET-UIO66-1. On the whole, the experimental results clearly indicated that UIO-66 could act as a nucleating agent to significantly up-regulate the crystallization rate and crystallization temperature of PET. Furthermore, the effect of metal–ligand coordination on PET and other polyesters due to MOFs’ defects is worth studying in depth.

## Figures and Tables

**Table 1:** Thermal behaviors of PET and PET/UIO-66 nanocomposites obtained from DSC.

Samples	1st Heating					Cooling	
	*T*_g_ (°C)	*T*_cc_ (°C)	Δ*H*_cc_ (J/g)	*T*_m_ (°C)	Δ*H*_m_ (J/g)	*T*_c_ (°C)	Δ*H*_c_ (J/g)
PET	76.6	135.6	26.9	252.5	37.2	194.3	52.1
PET-UIO66-0.5	72.3	132.5	29.5	249.6	35.5	209.8	53.4
PET-UIO66-1	72.6	129.5	27.5	249.3	29.4	211.6	53.1
PET-UIO66-3	71.4	128.5	27.0	249.5	37.5	210.4	51.6

**Table 2:** Thermal behaviors of PET and PET/UIO-66 nanocomposites obtained from second heating scan.

Sample	*T*_m1_ (°C)	*T*_m2_ (°C)	Δ*H*_m_ (J/g)	χ_c_ (%)
PET	-	249.7	46.8	33.4
PET-UIO66-0.5	240.9	250.6	53.5	38.4
PET-UIO66-1	238.6	249.7	49.5	35.7
PET-UIO66-3	237.3	249.1	48.1	35.4

**Table 3:** Thermal behaviors of PET and PET/UIO-66 nanocomposites obtained from DSC.

Cooling Rate	PET		PET-UIO66-0.5		PET-UIO66-1		PET-UIO66-3	
	n	Z_c_	n	Z_c_	n	Z_c_	n	Z_c_
5	2.78	0.44	3.48	0.68	3.65	0.68	3.32	0.70
10	2.91	0.80	3.46	0.99	3.53	1.01	3.56	0.97
15	2.38	0.91	3.45	1.05	3.52	1.07	3.45	1.06
20	2.46	0.94	3.37	1.07	3.42	1.09	3.48	1.07

**Table 4:** Thermal stability of PET and PET/UIO-66 nanocomposites.

Sample	*T*_5%_ (°C)	*T*_10%_ (°C)	*T*_max_ (°C)	Residue (%)
PET	405.2	414.6	446.2	13.2
PET-UIO66-0.5	401.6	412.4	446.0	17.4
PET-UIO66-1	401.7	411.5	444.8	19.7
PET-UIO66-3	395.9	406.9	438.8	21.2

**Table 5:** Detailed tensile properties of PET and PET/UIO-66 nanocomposites.

Sample	Yield Strength (MPa)	Elongation at Break (%)
PET	41.2 ± 3.5	217.0 ± 27.8
PET-UIO66-0.5	50.0 ± 1.2	229.6 ± 33.5
PET-UIO66-1	51.9 ± 0.6	207.5 ± 43.5
PET-UIO66-3	57.6 ± 4.3	14.6 ± 8.3

## Data Availability

Data is contained within the article.
